# Supplementary material for: Intermittency in the not-so-smooth elastic turbulence
Source: Nat Commun. 2024 May 27;15:4070. doi: 10.1038/s41467-024-48460-5 (PMC11130217; doi:10.1038/s41467-024-48460-5)
Supplement: Supplementary file 1 — Supplementary Information [file 41467_2024_48460_MOESM1_ESM.pdf]

Supplemental Material: Intermittency in the *not-so-smooth* elastic turbulence

Rahul K. Singh,[1] Prasad Perlekar,[2] Dhrubaditya Mitra,[3] and Marco E. Rosti[1,*]

[1] *Complex Fluids and Flows Unit, Okinawa Institute of Science and Technology Graduate University, Okinawa 904-0495, Japan*
[2] *TIFR Centre for Interdisciplinary Sciences, Tata Institute of Fundamental Research, Gopanpally, Hyderabad 500046, India*
[3] *Nordita, KTH Royal Institute of Technology and Stockholm University, Hannes Alfvéns väg 12, 10691 Stockholm, Sweden*

SUPPLEMENTARY DISCUSSION

A. Energy Spectra and flux

We show several crucial aspects of our DNS.

1. **Elastic turbulence appears at De > 1**
   We show the energy spectrum $E(k)$ for the Newtonian and small De (=1/9, 1/3) flows in Fig. (S1a), which remain devoid of any appreciable scaling regime. Energy is concentrated in the largest scales and the energy per mode decays sharply as we go to small scales.

2. **Energy spectra in wavenumber and frequency domain shows same scaling exponent.**
   In Fig. (S1b), we plot the energy spectra, $E(f)$, of the time-series of velocity measured at a fixed Eulerian point, for De $= 1$. A clear scaling regime of $E(f) \sim f^{-4}$ spans more than a decade in frequencies f. This energy spectrum is obtained by applying a Hanning window to the velocity field time-series recorded at a single point, which is then averaged over numerous (128^2) such points in the flow domain.

3. **Results are independent of grid resolution.** We also show the independence of our results from the choice of grid resolution in Fig. (S1c), where the plots of fluid energy spectra for De $= 1$ from simulations of grid sizes $N = 512$ (blue) and $N = 1024$ (yellow) closely follow each-other.

4. **The advective nonlinearities are not responsible for ET**
   We show the spectra from a simulation where the nonlinear term was set to zero. This confirms that ET we observe is indeed sustained by purely elastic effects.

5. **Universality with respect to force.**
   We find that the spectral exponent remains unchanged for two different forcing scheme (random forcing and ABC). We also find the scaling exponents for Σ_2 and Σ_4 to be 2.94 ± 0.21 and 4.32 ± 0.27 respectively when a random forcing is used, which are well within the error bars of the exponents computed when the forcing was ABC.

6. **Universality with respect to model for polymer.** We also obtain the same exponent for energy spectra for the Oldroyd-B model, which we use in all the simulations in our paper, and the FENE-P model.

1. *Fluxes*

The contribution to the flux of kinetic energy from polymer stress, viscous stress, and advective nonlinearity are, respectively:

$$\mathcal{T}_\mathrm{p}(K) = \frac{\mu_\mathrm{p}}{\rho_\mathrm{f}\tau_\mathrm{p}} \int_0^K d\Omega\ k^2\ dk\ u_\alpha(\boldsymbol{k}) k_\beta C_{\alpha\beta}(-\boldsymbol{k}), \tag{S1a}$$

$$\mathcal{D}(K) = \frac{\mu_\mathrm{f}}{\rho_\mathrm{f}} \int_0^K d\Omega\ k^2\ dk\ k^2 u_\alpha(\boldsymbol{k}) u_\alpha(-\boldsymbol{k}), \tag{S1b}$$

$$\Pi(K) = i \int_0^K d\Omega\ k^2\ dk\ u_\alpha(-\boldsymbol{k}) \int d\boldsymbol{q}\ q_\beta u_\beta(\boldsymbol{q}) u_\alpha(\boldsymbol{k}-\boldsymbol{q}). \tag{S1c}$$

* marco.rosti@oist.jp

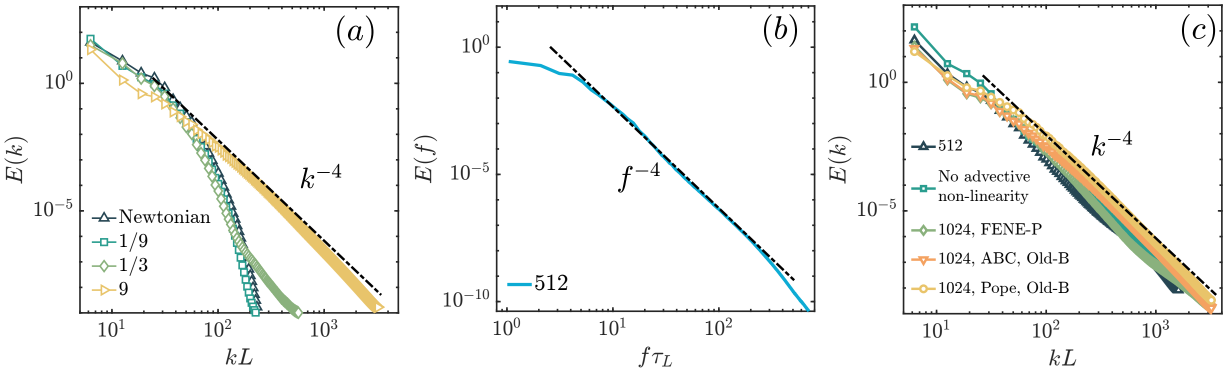

FIG. S1. **Energy spectra.** (a) Energy spectra for Newtonian and small De falls sharply – do not show any scaling behavior. This is compared against that for De $= 9$, where the spectrum decays as k^{-4} (see also Fig.3 of main text). (b) The temporal energy spectrum shows a similar $E(f) \sim f^{-4}$ power–law behaviour (computed for a smaller grid size $N^3 = 512^3$). (c) Universality with respect to grid size, polymer model, and the forcing scheme. The same k^{-4} scaling is obtained for simulations with smaller grid size $N^3 = 512^3$, no advective non-linear term, FENE-P model of polymers, a different forcing scheme [1] –the force is δ-correlated in space and exponentially correlated in time. For reference, we also show the k^{-4} spectrum for our $N^3 = 1024^3$ simulation with the non-linear term using the Oldroyd-B model of polymers excited by the ABC forcing scheme (see main text).

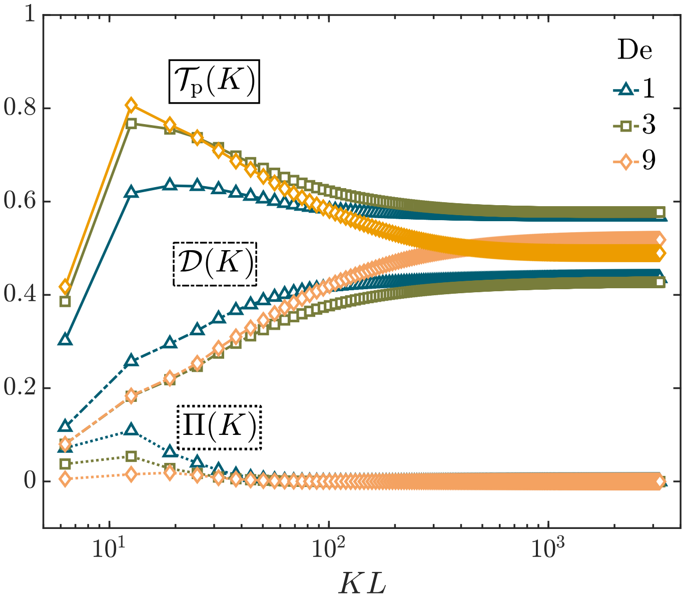

FIG. S2. **Energy fluxes.** The polymeric contribution $\mathcal{T}_{\rm p}(K)$ to the energy transfer to smaller scales compared to the fluid dissipation $\mathcal{D}_{\rm f}(K)$ for all three De numbers. Note the near zero contribution of the nonlinear advective flux for $KL \geq 30$.

We plot them in Fig. (S2). The fluid dissipation and polymer contributions are, as expected, comparable over a wide range of scales while the contribution from the advective nonlinear is almost zero (for $KL \geq 30$). This justifies the dominant balance used in the main text.

2. *Scaling argument*

Let us first state a well–known result [see, e.g., 2, section 4.5] for the spectrum of a statistically stationary and isotropic random vector function $\boldsymbol{w}(\boldsymbol{x})$. We define

$$\Gamma(r) \equiv \langle w_\alpha(\boldsymbol{x}) w_\alpha(\boldsymbol{x}+\boldsymbol{r}) \rangle \,, \tag{S2}$$

where $r = |\boldsymbol{r}|$. Here we have assumed that the statistics of $\boldsymbol{w}$ are isotropic, hence $\Gamma(r)$ is a function of r alone, not $\boldsymbol{r}$. In this case, the Wiener–Khinchin formula for the spectrum, $G(k)$, of $\boldsymbol{w}$ is:

$$G(k) = \frac{1}{\pi}\int_0^\infty kr\Gamma(r)\sin(kr)dr. \tag{S3}$$

If, in addition, $\boldsymbol{w}$ is scale invariant with a scaling exponent h, then under scaling $x \to \lambda x$ we obtain

$$\boldsymbol{w} \to \lambda^h \boldsymbol{w}, \quad \Gamma \to \lambda^{2h}\Gamma, \quad k \to \frac{1}{\lambda}k. \tag{S4}$$

As $\boldsymbol{w}$ is scale invariant, its spectrum must be a power-law, hence we have

$$G(k) \sim k^{-n}. \tag{S5}$$

Substituting (S4) and (S5) to (S3) we obtain

$$2h + 1 = n. \tag{S6}$$

Strictly speaking, this result holds only for $1 < n < 3$. For n outside this range, (S6) still holds under the following conditions:

1. $G(k)$ shows power-law scaling with a range of Fourier modes $k_0 < k < \Lambda$ where k_0 and Λ are the infra-red cutoff and ultra-violet cutoff, respectively.
2. Outside these cutoffs, $G(k)$ goes to zero fast enough such that $\int_0^\infty dkG(k)$ is finite.

Typically, these conditions are satisfied by all hydrodynamic quantities. Thus, there is a range of scale $(1/\Lambda) > r > (1/k_0)$ over which $\boldsymbol{w}(r)$ is scale-invariant with a scaling exponent h that satisfies (S6). Also note that the second order structure function of $\boldsymbol{w}$

$$S_2(r) = \left[\{\boldsymbol{w}(\boldsymbol{x}+\boldsymbol{r}) - \boldsymbol{w}(\boldsymbol{x})\}\cdot\left(\frac{\boldsymbol{r}}{r}\right)\right]^2 = \frac{2}{3}\left[\Gamma(0) - \Gamma(r)\right] \propto \Gamma(r) \sim r^{2h} \sim r^{n-1}. \tag{S7}$$

Let us now consider elastic turbulence where the velocity field is scale invariant with a scaling exponent h and the tensor $C_{\alpha\beta}$ is scale invariant with a scaling exponent $2b$. This implies that the tensor $\mathcal{B}$ is scale invariant with scaling exponent b. In other words, under rescaling $x \to \lambda x$,

$$\boldsymbol{u} \to \lambda^h \boldsymbol{u} \quad \text{and} \quad B_{\alpha\beta} \to \lambda^b B_{\alpha\beta}. \tag{S8}$$

Let us now assume

$$E(k) \sim k^{-\xi} \quad \text{and} \quad E_{\rm p}(k) \sim k^{-\chi}. \tag{S9}$$

Applying (S6) we obtain

$$2h + 1 = \xi \quad \text{and} \quad 2b + 1 = \chi. \tag{S10}$$

Note that (S6) can be extended to apply to the second rank tensor $\mathcal{B}$ in a straightforward manner. In ET, the advective term in the momentum equation is small (because Re is small) and for small scales the external force in zero. Hence at small scales, statistical stationarity implies that we expect the dominant balance to be

$$2\mu_{\rm f} S_{\alpha\beta} \sim \frac{\mu_{\rm p}}{\tau_{\rm p}} C_{\alpha\beta} \tag{S11}$$

where $S_{\alpha\beta} = \frac{1}{2}(\partial_\alpha u_\alpha + \partial_\beta u_\beta)$. Applying scale invariance, (S8) to (S11), we obtain

$$h - 1 = 2b. \tag{S12}$$

Finally, substituting (S10) in (S12) we obtain

$$\xi = 2\chi + 1. \tag{S13}$$

B. Structure Functions

The usual structure functions defined by the moments of first differences of velocity are:

$$S_{\mathrm{p}}(r) \equiv \langle |\delta u(\boldsymbol{r})|^p \rangle, \quad \text{where} \tag{S14a}$$

$$\delta u(\boldsymbol{r}) \equiv [u_\alpha(\boldsymbol{x}+\boldsymbol{r}) - u_\alpha(\boldsymbol{x})] \frac{r_\alpha}{|\boldsymbol{r}|}. \tag{S14b}$$

From the main text we repeat the definition of the structure function of second differences

$$\Sigma_{\mathrm{p}}(r) \equiv \left\langle |\delta^2 u(\boldsymbol{r})|^p \right\rangle, \quad \text{where} \tag{S15a}$$

$$\delta^2 u(\boldsymbol{r}) \equiv [u_\alpha(\boldsymbol{x}+\boldsymbol{r}) - 2u_\alpha(\boldsymbol{x}) + u_\alpha(\boldsymbol{x}-\boldsymbol{r})] \left(\frac{r_\alpha}{|\boldsymbol{r}|}\right). \tag{S15b}$$

1. *Second order structure functions*

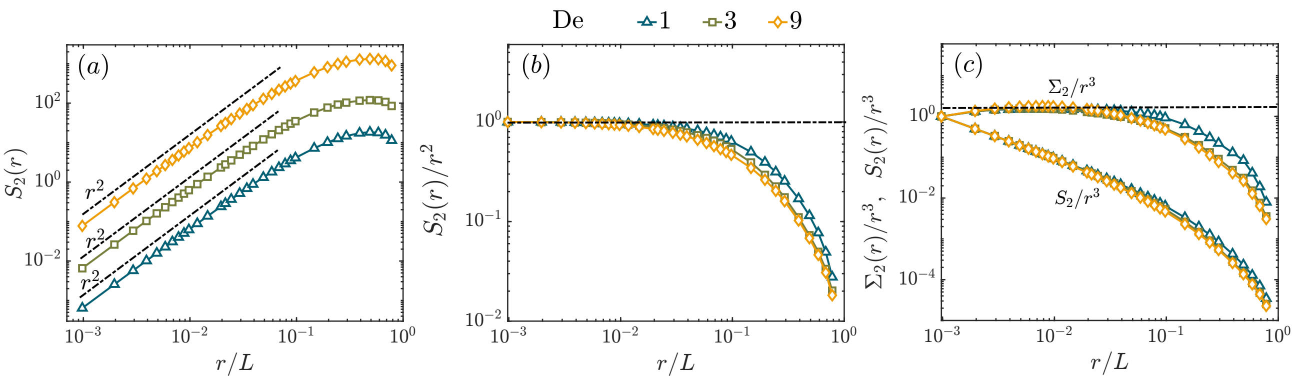

FIG. S3. **Second order structure functions** (a) The second order structure function $S_2(r)$ as a function of r, for De $= 1, 3$, and 9. (b) $S_2(r)/r^2$ for three different De ranging from 1 to 9. The range of analytic scaling $S_p \sim r^p$ decreases with increasing order of the moments as intermittent effects become more important. (c) $\Sigma_2(r)/r^3$ and $S_2(r)/r^3$ as a function of r. The range of analytic scaling (shown for S_4) remains independent of De.

In Fig. (S3a) we show the structure functions $S_2(r)$ for different De as a function of r on a log-log scale. We obtain the trivial scaling $S_2 \sim r^2$ as $r \to 0$. In Fig. (S3b) we plot $S_2(r)/r^2$ for three different De ranging from 1 to 9. At small enough r they all show $S_2 \sim r^2$. As r increases they all depart from this trivial scaling at a length scale ℓ which depends very weakly on De, if at all. Does the departure from trivial scaling shows a new scaling range? From Fig. (S3a) it is unclear if there is a scaling range at intermediate r. Now we turn to second order structure function of $\delta^2 u$, Σ_2. We find that $\Sigma_2(r)$ shows a significant scaling range as $r \to 0$ with the non-trivial scaling exponent $\zeta_2 \approx 3$, see the main text. To substantiate this further we plot in Fig. (S3c) Σ_2/r^3 and S_2/r^3. The former shows a plateau confirming $\Sigma_2 \sim r^3$, while the latter shows practically no plateau. There is no range of scales where the scaling $S_2 \sim r^3$ is obtained.

2. *Correlation function*

Note that two–point correlation function of velocity $C(r) \equiv \langle \delta u(r) \delta u(-r) \rangle$ is related to Σ_2 by

$$\Sigma_2(r) = \left\langle [\delta u(r) + \delta u(-r)]^2 \right\rangle = 2S_2(r) + 2C(r). \tag{S16}$$

Then the consequence of our results is that:

$$2C(r) = \Sigma_2(r) - 2S_2(r) \sim Ar^3 - Br^2, \tag{S17}$$

where A and B are two constants. Hence in the limit $r \to 0$, $C(r) \sim r^2$. We check this explicitly by plotting $C(r)/r^2$ as a function of r on a log-lin scale in Fig. (S4) for three De. At small r, the plot becomes flat, confirming our expectations.

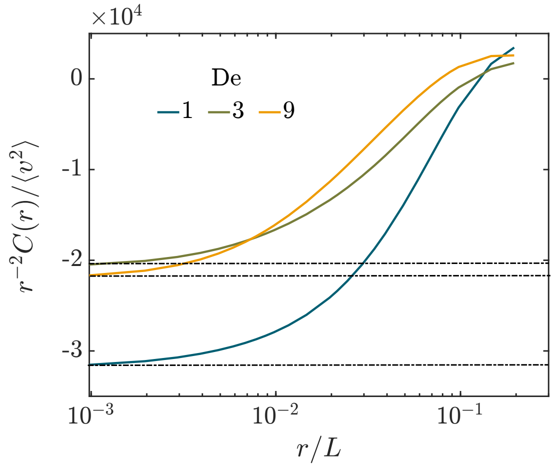

FIG. S4. **Correlation of velocity fluctuations** The semilog plot of the normalized, compensated correlation $C(r) \equiv (\Sigma_2(r) - 2S_2(r))/2 = \langle \delta u(r) \delta u(-r) \rangle$. This correlator gets its dominant contribution from $S_2(r)$ in the limit $r \to 0$, thus recovering the analytic behaviour.

Another way to see this is that, we expect the velocity gradients to be smooth for small r, whereby $u_\alpha(\boldsymbol{x}+\boldsymbol{r}) - u_\alpha(\boldsymbol{x}) \equiv \delta u_\alpha(r) \sim r_\alpha G_{\alpha\beta}$ and $\delta u_\alpha(-\boldsymbol{r}) \sim -r_\alpha G_{\alpha\beta}$, where $G_{\alpha\beta}$ is the gradient of the velocity field evaluated at $\boldsymbol{x}$. Consequently, $C(r) \sim -r^2$ and $S_2(r) \sim r^2$ have the same leading order behavior, and neither reveals the subdominant non-trivial scaling.

3. *Higher order structure functions*

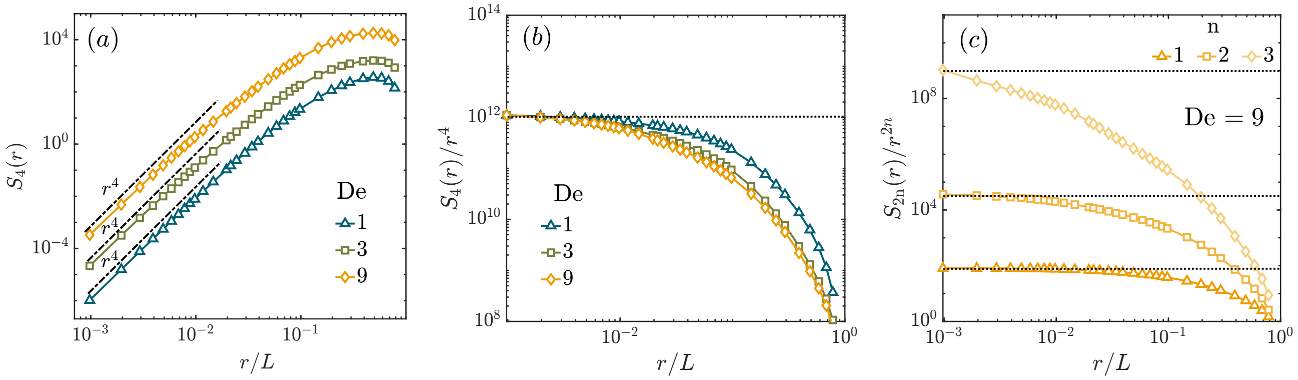

FIG. S5. **Higher order structure functions** (a) The fourth structure function $S_4(r)$ as a function of r, for De $= 1, 3$, and 9. We obtain the trivial scaling $S_4(r) \sim r^4$ as $r \to 0$. (b) $S_4(r)/r^4$ for three different De ranging from 1 to 9. (c) $S_{2\mathrm{n}}/r^{2n}$ as a function of r for De $= 9$ for $n = 1, 2$ and 3. The range over which the trivial scaling is valid decreases as we consider structure functions of higher order.

In Fig. (S5a) we plot the fourth structure function $S_4(r)$ as a function of r, for De $= 1, 3$, and 9. We obtain the trivial scaling $S_4(r) \sim r^4$ as $r \to 0$. In Fig. (S5b) we plot $S_4(r)/r^4$ for three different De ranging from 1 to 9. We find that all of them depart from trivial scaling at large r, but the scale at this this departure appears is almost independent of De. In Fig. (S5c) we plot the even order structure functions $S_{2\mathrm{n}}/r^{2n}$ as a function of r. As $r \to 0$, $S_{2\mathrm{n}}(r) \to r^{2n}$. Thereby we confirm, following the prescription in Ref. [3], that the structure functions of all order are analytic. The structure functions begin to depart from this analytic scaling at a scale that depends very weakly on De (if at all), but this scale decreases as p increases. The same behaviour was observed in Ref. [3] for the case of Newtonian HIT.

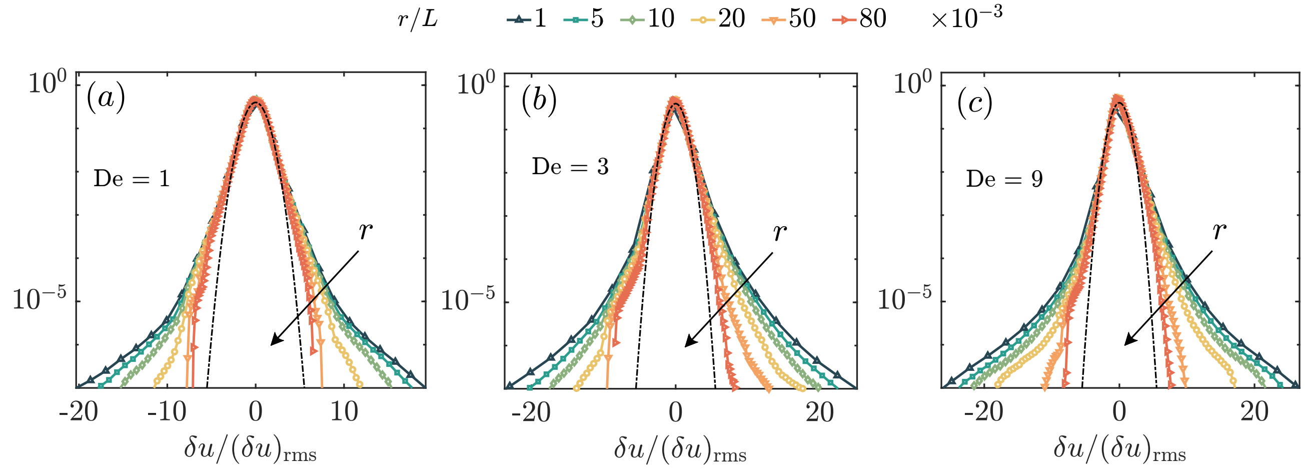

FIG. S6. **Probability distributions of $\boldsymbol{\delta u}$** The PDF of δu (normalized by their root–mean–square value) for different values of r, for: (a) $\mathrm{De} = 1$, (b) $\mathrm{De} = 3$, and (c) $\mathrm{De} = 9$. For comparison, we have plotted an Gaussian distribution as a dashed black line.

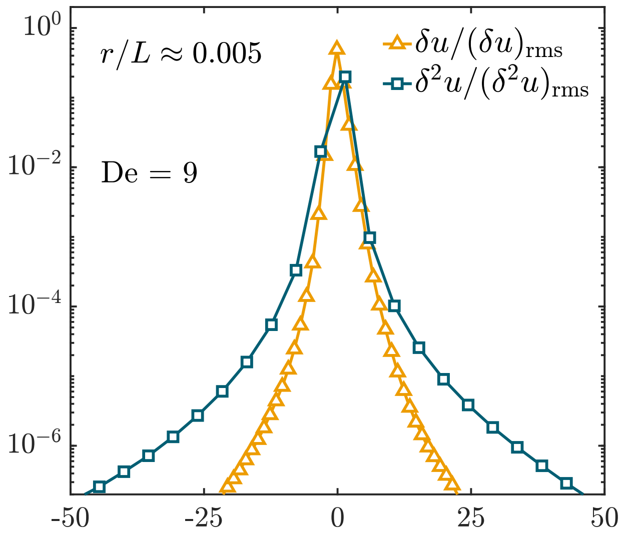

FIG. S7. **Intermittency in velocity differences** The PDF of $\delta^2 u(r)$ and $\delta u(r)$ for a representative value of r in the scaling range. The tail of the PDF of second difference falls off much slower than that of the first difference. This clearly indicates that the second differences are more intermittent than the first.

C. Probability distribution function of velocity differences

An evidence of intermittency is non-Gaussian behaviour of the tail of the PDF of velocity differences across a length scale. In the main text we show the PDF of second difference of velocity, $\delta^2 u$ across a length scale r. These PDFs are non-Gaussian if the scale r falls within the scaling range, $r/L \gg 1$, of the structure functions. In Fig. (S6) we plot PDF of δu for several different values of r for the three values of De. These PDFs are non-Gaussian too. But they are less intermittent than the corresponding PDF of the second difference of velocity $\delta^2 u$, see Fig. (S7) where we plot the two PDFs for a fixed representative value of r within the scaling range. This demonstrates what we have already commented on, the intermittency is a fundamental property of the velocity difference, but it is best revealed by the second difference, $\delta^2 u$.

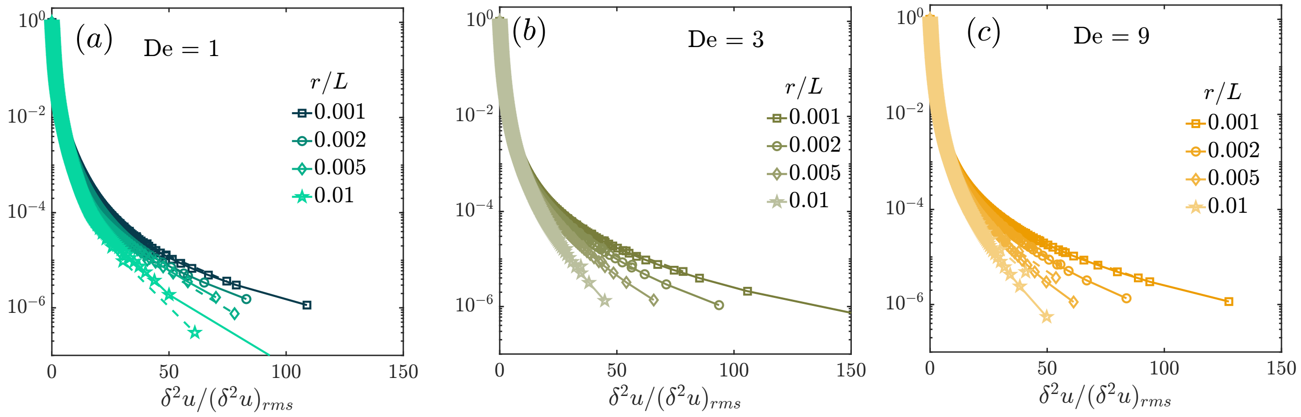

FIG. S8. **Complementary Cumulative Distribution** CCD functions of x component of $\delta^2 u$ (normalized by their root-mean-square value) for (a) De = 1, (b) 3, and (c) 9. The solid and dashed curves correspond respectively to CCDs for F^+ and F^-, calculated by rank-order method.

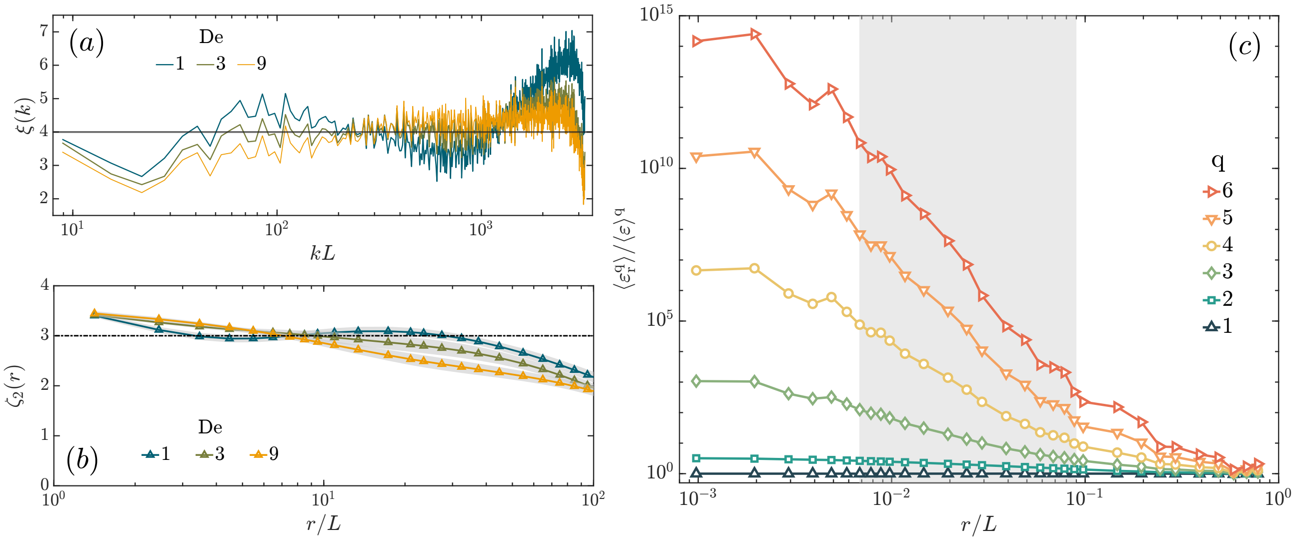

FIG. S9. **Exponents of the power-law scalings** (a) The local slopes of energy spectra for De $= 1, 3$, and 9 after a 3-point moving average. (b) The local slopes ζ_2 for $\Sigma_2(r)$ for all three De. The solid curves correspond to the mean value of the exponents with the standard deviation shown as shaded region. The statistics were obtained using 18 field snapshots. (c) The log-log plot of the scale averaged fluid energy dissipation rate $\langle \varepsilon_r^q \rangle$ versus the scale r for De = 3.

1. *Cumulative probability distribution*

We now consider two complementary cumulative probability distribution (CCD) functions

$$F^+(X) = 1 - \int_0^X P(x)dx \quad \text{and} \quad F^-(X) = 1 - \int_{-X}^0 P(x)dx, \tag{S18}$$

where X is positive and $P(x)$ is the probability distribution function of x. The two CCDs characterise the positive and negative tails of the PDF respectively. In Fig. (S8) we plot them, $F^{\pm}(\delta^2 u)$, for the second differences of velocity. The solid lines correspond to the CDFs computed for $\delta^2 u \geq 0$, while the dashed curves correspond to CDFs for $\delta^2 u \leq 0$. The CDFs are calculated using rank-order method [4], thereby they are free of binning errors that plague the usual PDFs that are calculated from histogram.

D. Computing Exponents

In this section, we detail the computation of scaling exponents of structure functions and energy spectra in terms of local slopes of the log-log curves. These have been shown in panels (a) and (b) of Fig. (S9). We show in panel (c) the scale-averaged energy dissipation rate $\langle \varepsilon_r \rangle$.

The scaling exponents ξ of the energy spectrum $E(k)$ shown in panel (a) are the 3-point moving averages of their local slopes. The mean exponents and their standard deviations are then found to be $-4.0 \pm 0.6, -4.0 \pm 0.3$, and -4.0 ± 0.4 for $\mathrm{De} = 1, 3$, and 9 respectively. Similarly, the local slopes ζ_2 for the second-order second difference structure functions $\Sigma_2(\mathrm{r})$ are plotted in panel (b). We compute these local exponents for 18 different time-snapshots. The set of 18 exponents for each r is then used to compute the local mean and deviation. We plot the mean values as a solid curve and show the corresponding deviation as shaded regions. The expected value of 3 is marked by a dash-dotted line for reference.

Finally, we show in panel (c) the log-log plots of the integer moments q of the scale-averaged fluid energy dissipation rate $\langle \varepsilon_r^q \rangle$ versus the scale r (for $\mathrm{De} = 3$). The emergence of a clear power-law regime (for $7 \times 10^{-3} \lesssim r/L \lesssim 9 \times 10^{-2}$) enables us to compute the multifractal spectrum (plotted in Fig.5 of the main text) using $q \in [-6, 6]$.

SUPPLEMENTARY REFERENCES

[1] V. Eswaran and S. Pope, Computers and Fluids **16**, 257 (1988).
[2] U. Frisch, *Turbulence the legacy of A.N. Kolmogorov* (Cambridge University Press, Cambridge, 1996).
[3] J. Schumacher, K. R. Sreenivasan, and V. Yakhot, New Journal of Physics **9**, 89 (2007).
[4] D. Mitra, J. Bec, R. Pandit, and U. Frisch, Physical review letters **94**, 194501 (2005).
